# Understanding how shared decision‐making approaches and patient aids influence patients with advanced cancer when deciding on palliative treatments and care: A realist review

**DOI:** 10.1111/hex.13822

**Published:** 2023-07-13

**Authors:** Michelle Edwards, Daniella Holland‐Hart, Mala Mann, Kathy Seddon, Peter Buckle, Mirella Longo, Anthony Byrne, Annmarie Nelson

**Affiliations:** ^1^ Division of Population Medicine, Marie Curie Palliative Care Research Centre Cardiff University Cardiff Wales UK; ^2^ Marie Curie Palliative Care Research Centre Wales Cancer Research Centre Cardiff Wales UK

**Keywords:** advanced cancer, decision support, palliative care, palliative treatment

## Abstract

**Background:**

Patients with advanced incurable cancer face difficult decisions about palliative treatment options towards their end of life. However, they are often not provided with the appropriate information and support that is needed to make informed decisions. This review aimed to identify contexts and mechanisms associated with communication tools, patient decision‐aids and shared decision‐making (SDM) approaches that influence patient outcomes.

**Methods:**

We used a realist review method to search for published studies of patients (adults > 18) with advanced cancer who were expected to make a decision about palliative treatment and/or supportive care in consultation with healthcare practitioners. We appraised and synthesised literature describing the contexts of (when and how) decision aids and SDM approaches are used, and how these contexts interact with mechanisms (resources and reasoning) which impact patient outcomes. Stakeholders including academics, palliative healthcare professionals (HCPs) and people with lived experience of supporting people with advanced incurable cancer contributed to identifying explanatory accounts. These accounts were documented, analysed and consolidated to contribute to the development of a programme theory.

**Results:**

From the 33 included papers, we consolidated findings into 20 explanatory accounts to develop a programme theory that explains key contexts and mechanisms that influence patient and SDM. Contexts include underlying patients' and HCPs' attitudes and approaches. These need to be understood in relation to key mechanisms, including presenting information in multiple formats and providing adequate time and opportunities to prepare for and revisit decisions. Contexts influenced mechanisms which then influence the levels of patient decisional satisfaction, conflict and regret.

**Conclusions:**

Our programme theory highlights mechanisms that are important in supporting shared treatment decisions for advanced noncurative cancer. The findings are informative for developing and evaluating interventions to improve understanding and involvement in SDM for patients with advanced incurable cancer.

**Patient and Public Contribution:**

We included patient and public involvement (PPI) representatives in four stakeholder meetings. PPI helped to define the scope of the review, identify their unique experiences and perspectives, synthesise their perspectives with our review findings, make decisions about which theories we included in our programme theory and develop recommendations for policy and practice and future research.

## 1 BACKGROUND

Patients with advanced incurable cancer are typically offered systemic treatments with palliative intent, that is, chemotherapy, and immunotherapy and/or radiotherapy. These treatments can kill cancer cells to improve symptom control and change the course of the disease to extend life^1^ Patients can also be offered palliative or supportive care (which focuses on other forms of pain relief, symptom management and taking care of psychological and spiritual needs).^2^ However, there exists a balance between the moderate benefit of palliative chemotherapy and the burden of treatment.^3^, ^4^, ^5^ Palliative systemic treatment can relieve some symptoms, enhance the quality of life and optimise symptom control,^6^, ^7^ when used near the end of life. However, it can also result in detrimental effects, such as increased toxicities, worsening quality of life,^4^, ^8^ increased treatment‐related mortality,^9^, ^10^ increased hospital admissions^11^ and unnecessary cost to the healthcare system.^12^ Studies have shown that up to 17.4% of patients treated with palliative chemotherapy in the last month have died within 30 days of starting treatment.^12^, ^13^

When delivered early (90 days before death), a palliative/supportive care‐only approach can help reduce symptom burden and improve mood and quality of life for patients^14^, ^15^ and provide improvements to caregiver quality of life, lower caregiver burden^16^ and better preparedness for caregiving.^17^ A Lancet report on the value of death, proposes that earlier referral to palliative care services, the use of advanced care plans, decision support tools, health communication strategies and care pathways are also important to reduce levels of overtreatment.^18^ Furthermore, there is strong evidence of the cost‐effectiveness of palliative care only as an option.^19^

It is imperative that all treatment and alternative care options are presented to patients so that they can be fully informed about the potential benefits and harms of treatments and alternative options before making treatment decisions. However, decisions regarding whether to start, continue palliative treatment or accept supportive and palliative care are complex for healthcare professionals (HCPs) and patients, as individual responses to treatments vary, are difficult to predict, and there can be uncertainty about prognosis.^18^ Personal circumstances including health status, social circumstances and preferences for quality of life towards the end of life need to be carefully considered and incorporated into treatment and care decisions.^19^

The National Institute of Clinical Excellence's guidance on improving supportive and palliative care for adults with cancer recommends that patients should be involved in decisions about their treatment and care along the cancer pathway using a shared decision‐making (SDM) approach.^2^ SDM is a two‐way exchange of information and treatment preferences, involving a HCP and a patient. Stiggelbout et al.^20^ have described four steps of SDM: (1) where the HCP tells a patient that a decision is to be made and their opinion is important, (2) the HCP explains options and pros and cons, (3) HCP and patient discuss patient's preferences and HCP supports patient to deliberate and (4) HCP and patient discuss patients preference for involvement in decision and make or defer decision, and discuss follow‐up.

Decision support tools (decision aids) can be used to provide information about the risks and benefits of treatments and enable patients to reflect on and communicate their preferences to HCPs and participate in SDM.^21^ Communication tools (such as question prompt lists) support patients to ask relevant questions in healthcare settings that can enhance their understanding of treatment and facilitate their involvement in decision making. However, recent research in lung cancer services has identified that there can be gaps in HCP communication around prognosis and missed opportunities for patients with advanced to engage with information and SDM, and decision support tools were not routinely used in practice.^19^

### 1.1 Aim

Our aim was to use a realist approach to understand how SDM approaches and patient aids influence patients with advanced cancer when deciding on palliative treatments and care.

We aimed to identify recent literature on SDM, decision support tools and communication tools used for all types of advanced cancer and to develop a programme theory to explain their impact on patients' experiences of treatment decision‐making.

Realist research typically seeks to answer ‘what works, for whom, under what circumstances?’, and ‘How?’, ‘Why?’ and ‘To what extent?’ does an intervention produce certain outcomes? Realist research focuses on developing theories to explain how different contextual factors influence mechanisms (related to resources put in place or the way that people reason) and how they might lead to different or similar outcomes.^22^, ^23^

Realist methods involve developing Context, Mechanism and Outcomes theories. This involves identifying how the different *contexts* (*HCP and patient circumstances or healthcare service delivery*) in which our intervention of interest (SDM approaches, and decision support tools and communication tools are used with patients with advanced and incurable cancer). Then, identify how contexts influence *mechanisms* (*intervention resources*, e.g., early integration of palliative care and *HCPs or patients reasoning*, e.g., preparedness for involvement in treatment decision‐making) to impact patient experience *outcomes*, such as patient's perceived quality of their decision‐making experience and satisfaction with their decision.

This realist review is registered on PROSPERO 2021 CRD42021251690.

## 2 METHODS

### 2.1 Realist review method

We used realist review methodology to identify, appraise and synthesise diverse forms of evidence to generate theories about how patient aids and SDM approaches are used and what outcomes are achieved.^23^ We drew on approaches used by Pawson and Tilley^22^ and Saul et al.^24^ to include the following steps: 1—define the scope of the review; 2—develop initial theories; 3—undertake evidence search, selection and appraisal; 4—data extraction; 5—analysis; 6—data synthesis using a framework; 7—develop narrative and make recommendations. While our original plan was to work within the timeframe of a typical rapid realist review, the review took longer than planned and we updated our search in November 2022 (see search strategy below). Thus, we have aligned our method with a realist review.

#### 2.1.1 Stakeholder involvement

In line with realist review methodology we worked collaboratively with stakeholders (e.g., practitioners, policy makers, patient representatives) to produce findings that can be translated to service improvements and intervention development.^24^, ^25^ We engaged with two patient and public involvement (PPI) representatives with experience in caring for a spouse with advanced cancer (K. S. and P. B.). This ensured that we were carrying out our research and making recommendations, which incorporated relevant first‐hand experiences of people who had supported relatives with advanced cancer towards the end of life. We also involved HCPs (oncology and palliative care) and experienced cancer and palliative care, and systematic review researchers at the start of the review to define its scope (step 1), develop and refine review questions and discuss the potential utilisation and relevance of findings [Stakeholder/PPI meeting 1].

#### 2.1.2 Initial literature scoping and programme theory

To help define the scope of the review and identify evidence to develop initial theories (step 2), we carried out a brief initial literature scoping exercise (step 1). We searched the literature using google scholar and were also recommended papers by our review team and stakeholders. Findings were extracted from studies about palliative treatment decision‐making (see Supporting Information: Appendix 1 for a list of papers).

#### 2.1.3 Initial theories (step 2)

**Figure 1:**
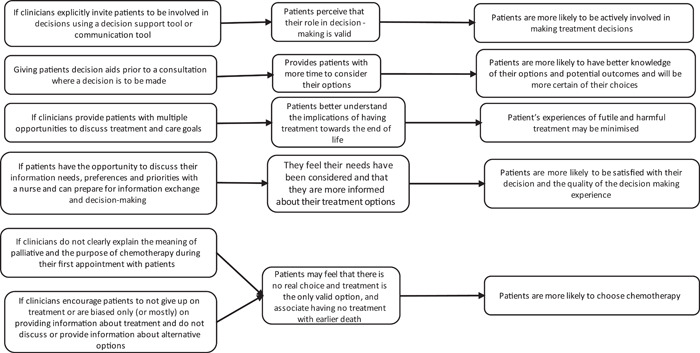
Initial programme theory.^25^

We generated a list of six initial theories using context, mechanism and outcome configurations (see example in Box 1). The initial theories highlight that HCP communication was an overarching *context*. Providing a balance of information about options, timing and support with decisions were important *contextual factors* (*c*) which influenced *mechanisms* (*m*) such as patient's attitudes and understanding about choice, their role in decision making, and how informed and prepared they felt. In turn, these influenced *outcomes* (*o*) related to patients' level of involvement, their choices, their satisfaction with their choices and the consequences of their choices (e.g., avoiding futile treatment near the end of life). We have illustrated our preliminary theories in our initial programme theory below (see Figure 1).

Box 1 **Example of a context, mechanism and outcome configuration**

If clinicians invite patients to make decisions using decision support or communication tools (c), patients may perceive they have a valid role in decision‐making (m) and are more likely to be actively involved in decision‐making (o).

#### 2.1.4 Literature search (step 3)

We included studies of patients (adults > 18) with advanced cancer (all cancers) who were offered and expected to make a decision about noncurative treatment (with a focus on chemotherapy, immunotherapy, radiotherapy) and/or supportive care in consultations with healthcare practitioners. We also included studies about patients who were making decisions about ending chemotherapy. Our interventions of interest were patient aids, communication tools, question prompt lists, web‐based interventions, SDM approaches that support or contribute to informed decision‐making and patient participation in decision‐making about noncurative treatment. Our outcomes of interest were related to patient's experiences (e.g., satisfaction with decisions, decisional regret, decision quality, satisfaction with information provided, desire for patient involvement, ability to engage with the decision‐making process).

#### 2.1.5 Search strategy

Search strategies were developed and searches were carried out by M. M. This initially included studies published from 2000 to April 2021 using the following databases: CINAHL (EBSCO), Cochrane Central (WILEY), Cochrane Library (WILEY), Embase (OVID), MEDLINE (OVID)PsycINFO (OVID), Social Science (PROQUEST) (see Supporting Information: Appendix 2 for search strategy). In addition, we searched ClinicalTrials.gov (www.clinicaltrials.gov) and World Health Organisation (WHO) International Clinical Trials Registry Platform (www.who.int/ictrp/en) for ongoing trials. M. E. and D. H.‐H. checked reference lists from primary studies and relevant systematic reviews were checked for further potentially relevant studies and carried out backward and forward citation tracking. Grey literature searches were carried out on Google Scholar and Google, University's Option Grid and the National Institute for Health and Care Excellence Evidence, UK National Health Websites and national and international government websites. We carried out an updated search in November 2022 using the same search strategy in Supporting Information: Appendix 2 to cover a lapse in time while this paper was being drafted.

All references were exported from the database to endnote 9 (Clarivate Analytics). Two reviewers (M. E. and D. H.‐H.) independently screened all the references by title and abstract to find articles that met our inclusion criteria (using yes‐include, maybe‐include or no‐exclude). We created an abstract and full‐paper screening tool to screen for inclusion and rank each paper selected as ‘yes’ or ‘maybe’ and from one to four in terms of relevance to our review questions and initial programme theory (highly, probably, possibly relevant or likely irrelevant) (see Supporting Information: Appendix 3). All ‘likely irrelevant’ papers were excluded at this stage.

#### 2.1.6 Data extraction (step 4)

We created a data extraction tool (see Supporting Information: Appendix 4) to extract quantitative, qualitative and contextual data from each remaining paper. Qualitative analysis of this data was conducted to create ‘explanatory accounts’ to draw out contexts, mechanisms and outcomes (step 2). Explanatory accounts use ‘if’, ‘then’ and ‘because’ statements to help describe the enabling and constraining factors or *contexts (If)* that influence *mechanisms (because)* and lead to *outcomes (then)* (see example in Box 2).^25^ Articulating realist theories as explanatory accounts can help when extracting a large amount of data from the literature and also help with clarity when discussing realist findings with stakeholders. Explanatory accounts are also useful for integrating theory into complex interventions.^25^

Box 2 **Example of an explanatory account**

*If* clinicians explicitly invite patients to be involved in treatment decisions and use a decision support tool or communication tool, *then* patients may be more likely to become actively involved in treatment decision making, *because* they will perceive that they have a valid role in decision‐making.

In line with a realist review approach, we employed a heuristic approach to examine where the value of a study is assessed by how much it is able to enhance the programme theory, hence, there is no other quality assessment Thus, we excluded papers at this stage that lacked sufficient evidence to create theories during the data extraction process, for example, did not include enough relevant data to construct any explanatory accounts Two reviewers extracted data (M. E. and D. H.‐H.) and discussed explanatory accounts frequently to enable refinement and consistency of application. When all data extraction forms were completed, there were 274 explanatory accounts drawn up.

As part of our stakeholder involvement process, we discussed explanatory accounts that we identified. Then, we co‐developed an additional 16 explanatory accounts with HCPs (based on their experience of clinical practice) and PPI representatives (based on their experiences of being a carer for a patient with advanced cancer and their knowledge from their involvement in cancer and palliative care studies). The total number of explanatory accounts (*n* = 290). This enabled us to refine explanatory accounts and develop new ones [Stakeholder/PPI meeting 2].

#### 2.1.7 Analysis and synthesis of data (step 5)

We recorded explanatory accounts from our data extraction and those from our engagement with PPI representatives [developed in stakeholder/PPI meeting 2] in a table with a record of the source. We then assessed all accounts and relevance to the aims of the review and excluded 84 accounts. We then consolidated the remaining 206 accounts into 20 accounts while paying attention to whether these accounts/configurations or aspects of them were novel or similar and whether they sufficiently reflected the original explanatory account using Pawson's reasoning processes.^26^ We then converted the 20 consolidated accounts into context, mechanism and outcome configurations to help us refine the initial programme theory. Explanatory accounts were then organised into topic themes.

We met with PPI representatives and stakeholders at this point to present a summary of our analysis of papers included in the review and asked them to provide feedback on them in terms of validity and relevance to the research questions [Stakeholder PPI meeting 3].

### 2.2 Data synthesis (step 6)

Realist research uses middle‐range theories (social theories that explain social behaviour but lie between minor working hypotheses and a unified theory explaining all social behaviour) to iteratively test these theories and build an overall programme theory.^27^ This approach is helpful in making sense of interventions and programmes that are complex and have outcomes that are context dependent.^22^, ^25^ We applied a conceptual framework for individual and family end‐of‐life decision making by Kim et al.^27^ to our analysis to help interpret our findings. This explains the ways in which patients with advanced illness and their caregivers/or healthcare providers make complex decisions at the end of life (including noncurative cancer treatments). The underlying assumptions of this conceptual framework are that: (1) healthcare decision making occurs in the context of cultural and social expectations, as well as established healthcare systems; (2) desirable decision processes and decision outcomes depend on patient–family–provider interactions and (3) the decision making is cyclical and iterative where decision outcomes influence future decision process and outcomes.

## 3 RESULTS

**Figure 2:**
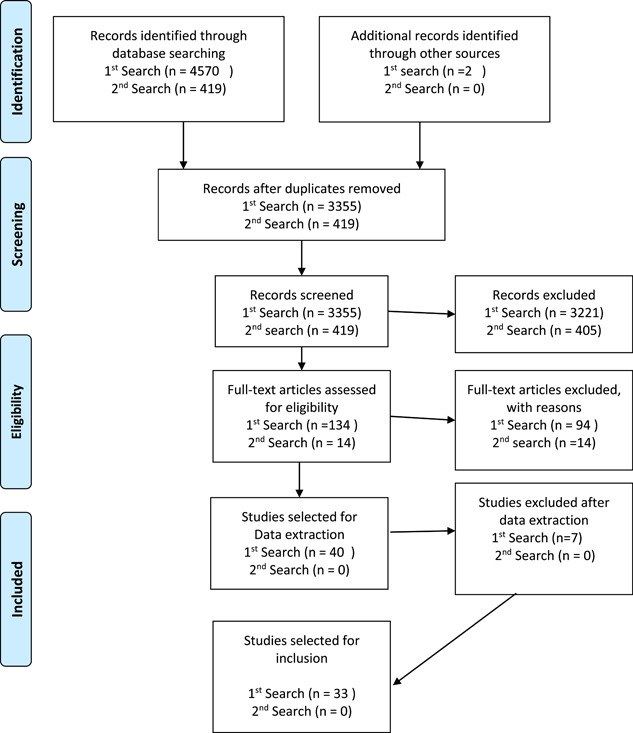
Search strategy and result.

The first search was carried out in June 2021. Records identified through the database and hand searches were *n* = 4570. After duplicates were removed (*n* = 3355), 3221 abstracts were excluded for not meeting the criteria and 134 full‐text papers were assessed for eligibility and *n* = 94 papers were excluded because they were ranked ‘likely irrelevant’. We selected 40 papers for data extraction based on our initial ranking of relevance. Then, seven were excluded after we identified that these papers did not contain enough relevant data to create theories. The final number of studies included in the first search of the review was 33, this was after an extensive search for explanatory accounts that were relevant to the review questions. Our updated search in November 2022 identified 419 records, after the removal of duplicates. We screened these records and read 14 papers in full that were relevant to the inclusion criteria. No new papers provided were included in the review. The results from search one and two and the screening process are outlined below in the Preferred Reporting Items for Systematic Reviews and Meta‐Analyses diagram (Figure 2).

From the 33 papers that we included, 20 papers referred to 18 specific interventions that have been utilised to support patients with advanced noncurative cancer with communication and decision‐making relating to anticancer palliative treatments (chemotherapy, immunotherapy or radiotherapy) and palliative care‐only decisions. One review paper on decision aids used for end‐of‐life decisions. Most papers discussed chemotherapy, one paper studied radiotherapy and one paper studied immunotherapy (see Table 1 below). A further 13 studies described using SDM approaches in relation to palliative systemic treatment (see Table 2 below).

**Table 1:** List of papers relating to 18 decision aids and communication tools.

Reference	Patient group	Decision context	Delivery method	When delivered
*Decision aids—video component*				
Dharmarajan et al.^28^	Advanced metastatic cancer	PRT/usual care	Hospitalised patients watched a video given to them on an iPad with a short section narrated by patient actors and a palliative care consultant	Before a PRT consultation during the patient's hospital stay
Bakitas et al.^29^	Gastrointestinal, breast cancer, genitourinary	Decision‐making about palliative treatments and advanced directives	Patients taking part in an education class given a 35 min DVD narrated by a social worker	Delivered before session 3 of a 6‐week education programme
	Haematological, advanced solid tumour, other solid tumour, haematological malignancy (prognosis 6–24 months)			
*Decision aids—audio component*				
Chiew et al.^30^	Hormone‐resistant metastatic breast cancer	First‐line chemotherapy treatment or best supportive care	Development and evaluation work with a researcher	Patients already had decided on their own treatment and were supporting research development
Fiset et al.^31^	Patients with stage IV non‐small cell lung cancer	Chemotherapy, radiotherapy, supportive care	The researcher gives the decision aid to the patient to take home	After an initial consultation and before a decision‐making consultation
Leighl et al.^32^	Advanced colorectal cancer	First‐line chemotherapy, supportive care, observation	An oncologist	Delivered in an initial consultation and patient takes away and bring back to next consultation to make a decision
Oostendorp et al.^33^	Advanced breast or colorectal cancer	Second‐line chemotherapy or supportive care	A nurse	A week after an initial consultation where an oncologist proposes treatment
Oostendorp et al.^34^ (same tool as above)	Advanced breast or colorectal cancer	Second line chemotherapy or supportive care	A nurse	A week after an initial consultation where an oncologist proposes treatment
Smith et al.^35^	Advanced breast, lung, colon, hormone‐refractory prostate cancer	Chemotherapy or supportive care	A graduate medical student (not involved in the patients' care)	Not clear, after a screening questionnaire and before a consultation
*Multicomponent*				
Jones et al.^36^	Metastatic castration‐resistant prostate cancer (life expectancy > 6 months)	Likely to be chemotherapy, hormonal therapy, immunotherapy or stopping treatment	Research nurse	Completed before consultation in a clinic visit and then discussed within the consultation at their next visit
Hollen et al.^37^ (same tool as Jones above)	Newly diagnosed breast cancer, advanced prostate cancer, advanced lung cancer	Likely to be chemotherapy, hormonal therapy, immunotherapy or stopping treatment	Health professionals (nurse and physician)	In an outpatient clinicUnclear of time intervals
Phillips et al.^38^	Some patients had stage IIIb–IV lung cancer (other patients with neurological and kidney disease)	Varied range of decisions about treatment and end‐of‐life care (not exclusive to palliative treatments)	Varied healthcare professionals in studies included in this review	At various times across studies included in the review
*Communication tools*				
Henselmans et al.^39^	Advanced cancer (beyond the first line of treatments) with a median life expectancy of <12 months	Disease‐targeted treatment (standard or experimental), best supportive care and watchful waiting	Mailed to patients	Before a scheduled appointment to discuss the start of a new line of treatment or disc
Bouleuc et al.^40^	Advanced cancer with a life expectancy of <12 months after their first consultation with the palliative care team	Chemotherapy, hormone therapy and radiotherapy, also taking a break or stopping treatment	Palliative care physicians gave the questionnaires to the patients at the end of each consultation	After their first consultation with the palliative care team
Clayton et al.^41^ (prognosis and end‐of‐life)	Advanced cancer receiving palliative chemotherapy	Focused on advanced care planning and end‐of‐life chemotherapy	QPL provided before the consultation	20–30 min before consultation with a palliative care clinician
Rodenbach et al.^42^	Advanced nonhaematologic cancer	Focuses on decisions about treatments and future care	Medical oncologists receive training for consultations. Social workers provided coaching for patients	Patients received a previsit individualised communication coaching session that incorporated a QPL
Walczak et al.^43^	Advanced cancer with prognosis <1 year	Seeking information regarding prognosis, EOL and future care and promoting discussion of advanced care planning	Nurse‐led communication programme including a QPL	Before, during and after consultations with an oncologist
Yeh et al.^44^	Metastatic head and neck cancer	A short form covering cancer treatment	Before their initial consultation at the clinic, patients received a copy of the QPL	Before initial consultation
*Web‐based interventions*				
DuBenske et al.^45^	Patients with advanced lung cancer and family caregivers	Facilitates communication between peers and learning about their treatment decision‐making experiences. Enables interaction with an expert for detailed questions	Interactive web‐based facilitates communication with the healthcare team	After the diagnosis of advanced cancer
Meropol et al.^46^	Patients with metastatic solid tumour	Generates more accurate expectations about the benefits and toxicities associated with treatment	Web‐based communication aid, online survey and assessment results reported to the clinician	Before consultations communication skills training (CST), plus a summary report to the physician or communication aid and CST without physician summary
*Consultation planning tool*				
Sepucha et al.^47^	Patients with curative and noncurative breast cancer	Decisions about surgery, adjuvant therapy, treatment for local recurrence, and metastatic disease.	Resource centre staff ‐trained facilitators without medical backgrounds	Planning sessions before consultations (in the breast clinics, facilitated by nurses, or community resource centres, facilitated by trained volunteers)

Abbreviations: EOL, end of life; PRT, palliative radiation therapy; QPL, question prompt list.

**Table 2:** List of 13 papers relating to studies of shared decision‐making approaches.

References (shared decision‐making approaches)	Patients with	Decision context
Alesi et al.^48^	Non‐small cell lung cancer	Discuss palliative care options
Back et al.^49^	Advanced cancer	Decision‐making about palliative anticancer therapy and phase 1 trials and advanced care planning
Clarke et al.^50^	Advanced solid tumours	Conversations after diagnosis of advanced cancer about withdrawal anticancer drugs
De Snoop‐Trimp et al.^51^	Glioblastoma	Whether to start second‐line (chemotherapy) treatment
Henselmans et al.^52^	Metastasised or inoperable tumours pancreas oesophagus, stomach, liver, gall bladder, bladder or sarcoma	The consultation was focussed on a decision about a new line of chemotherapy or the (dis)continuation of the current chemotherapy
Beaussant et al.^53^	Advanced solid cancer and haematological malignancies	Involvement in specific therapies' decision‐making
Bergqvist and Strang^54^	Metastatic breast cancer at least their second line of palliative chemotherapy	The decision to accept and continue palliative chemotherapy
Bruera^55^	Advanced breast cancer receiving palliative chemotherapy	First‐line chemotherapy or a median of 13 days after a second‐line chemotherapy consultation
Brom et al.^56^	Advanced cancer‐glioblastoma and metastatic colorectal cancer	Continuing chemotherapy
Chan et al.^57^	Metastatic cancer patients near end of life	Decisions on palliative anticancer therapy or aggressive supportive care
Koedoot et al.^5^	Metastatic cancer	The choice between palliative chemotherapy and the best supportive care
Nelson et al.^58^	Advanced lung cancer and family members	Palliative chemotherapy and supportive care
Sharma et al.^59^	Lymphoma/leukaemia, colorectal, breast, lung	Decisions centred on three topics: (1) disease‐modifying treatments; (2) hospice and (3) code status
	Noncolon gastrointestinal (e.g., pancreatic, liver), other (e.g., uterine, sarcoma)	

### 3.1 Realist theories

From our analysis and synthesis of all papers included, we created 20 consolidated theories using context mechanisms and outcome configurations. These explain patient‐related contexts which affect the decision‐making experiences, factors related to the presentation and format of decision aids, factors relating to timing and space for reflection, HCP‐related factors which affect communication and decision support and palliative care involvement in the process. The relationships between contexts (c) and mechanisms (m) and how they influence a range of different outcomes (o) for patients are reflected in the list of theories (see Table 3). The theories are then used to refine the initial programme theory.

**Table 3:** Realist theories.

Categories		Theories	Reference source (article number)
Patient knowledge, understanding, capacity to engage with information, prior experiences and information preferences	1	Patients' knowledge and understanding of the expertise, roles and aims of HCPs or the goals of decision aids and communication tools (C), can influence their perceptions of a realistic choice and role in decision‐making (M) and can mean that they do not see any treatment as a feasible option and may not express a desire for involvement in treatment decision making (O)	[32, 34, 39, 52, 53, 54]
	2	Cognitive abilities and level of education (C) can influence how patients engage with information in decision aids and consultations, as well as understand treatment goals and communicate with HCPs (M), which affects their ability to make informed decisions and motivation to actively participate in decision‐making (O)	[50, 51]
	3	Patients with previous knowledge and experience of cancer treatment (C), already have some understanding of the implications of treatment and are often comfortable engaging with detailed information in decision aids (including information on tumour response, the benefits and risks of palliative treatment options and survival) (M). Then, they may find receiving all relevant information about treatment options (included in consultations and decision aids) acceptable without a negative effect on their wellbeing and have a higher level of certainty about their decision or the level of control they feel they have about their decision (O)	[28, 34, 36, 37, 50]
	4	Previous knowledge and experience of treatments and side effects (C) can influence how patients interpret them in relation to cancer symptoms, their understanding and acceptance of the risk of side effects and their confidence in negotiating treatment options (M). This then can impact the value of a decision aid and influence treatment choices and patients' readiness to engage with their choice of treatment (O)	[28, 32, 34, 54, 60]
	5	When decision aids and tools are presented to them (C) most patients want accurate and honest information about their prognosis and all the relevant treatment options, side‐effects available to them (M), so that they can make appropriate decisions about starting, stopping or continuing treatments (O)	[32, 33, 36, 56]
	6	Some patients have different cultural preferences for engaging with information decision making that references end‐of‐life (C) and so patient aids and decision‐making approaches need to be adapted (M) to ensure patients are provided with accessible and culturally appropriate language and information to support their decision making (O)	[38, 61]
Presentation, content and format of decision aids	7	When patients with advanced cancer engage with multicomponent communication tools and decision aids to support decisions about palliative treatment and best supportive care (C), the multiple information sources help them develop skills and knowledge to gain a better understanding of potential treatment outcomes, risks and benefits of treatment options and clarification of their values and better communication with HCPs (M), which can influence a reduction in decisional conflict (O)	[31, 32, 45, 50, 51, 55, 61]
	8	When patients receive clear information in multiple formats within decision aids (C), this reinforces verbal information with written material that enhances patient comprehension and helps patients engage with information in different ways, (M) as it can improve their knowledge and understanding about treatment and communication with HCPs and can increase the satisfaction with the information and reduce decision conflict (O)	[31, 45, 49, 50]
	9	Patients who have been provided with decision aids that outline accurate and balanced information about all of their treatment options including best supportive care and likely outcomes (C), are more likely to have a more realistic understanding of survival chances (M) and be prepared to stop treatment (O)	[29, 31, 32, 45]
Timing of information, time and space for patients to engage with information (outside consultation), time discussing treatment options and alternatives in consultations	10	The timing of provision of decision and communication aids to patients with advanced cancer regarding treatment options for advanced cancer (C), such as offering the decision aid, as soon as possible after diagnosis can support patients by enabling them to process information, consider it with another person and enables time to prepare questions. Alternatively, it can overwhelm them because they are not ready for the information or when they realise their cancer is not curable and the nature of the decisions involved (M) and this can influence patients' acceptability of a decision aid, motivation to ask specific questions and their emotional reactions (O)	[31, 34, 36, 39, 40, 51]
	11	When patients with advanced cancer are given access to a patient aid to use at home before a consultation (where a decision is to be made) (C), the time between consultations to engage with a decision aid gives patients the opportunity to read through the decision aid and process the information and share and discuss the decision aid with others who can support them in their understanding and decision making (M). This influences patients' knowledge and understanding of prognosis, treatment options, risks and benefits of treatments and treatment goals and can reduce decisional conflict (O)	[32, 45, 50]
	12	The balance of time spent discussing symptoms and providing evidence‐based information about all of the risks and benefits of all potential treatments and alternative options (C) can affect patients' knowledge and expectations about the benefits of treatment options (M) and limit or create opportunities for patients to make informed decisions about treatments that suit their needs and preferences (O)	[48, 49, 56]
	13	If oncologists spend little or no time discussing alternatives to active treatments or explaining how treatment choices can affect patients' quality of life (C), then patients may not have a realistic expectation of palliative treatments and will not be adequately informed about supportive care as an option and how either option can have an impact on their life (M). So, they are not equipped to make fully informed decisions (O)	[48, 49, 56]
	14	If patients only have the opportunity to discuss their needs in relation to continuing treatments at routine appointments for evaluations and results (C) this can limit their opportunities to reflect on and evaluate their experiences of treatments in line with their expectations, preferences and goals along their cancer pathway (M) and can mean that patients can continue treatment without any re‐evaluation of their decisions (O)	[56]
Clinician's perception of their role, communication styles and approaches and the use of communication tools	15	When HCPs believe that their role is to make the final decision and/or that the patient does not have the knowledge or desire or responsibility to make a decision (M), then they are less likely to adopt a shared decision‐making approach. So, patients may defer their decision to the HCP (O), as they have not been provided with appropriate opportunities or knowledge or do not want to make the decision	[28, 31, 44]
	16	HCPs' communication approaches and the information that they present (e.g., information about prognosis), checking patients' information needs and understanding, endorsing a shared decision‐making approach, actively encouraging questions and their level of empathy (C), can influence patients' expectations of which treatments they might benefit from (M) and can have an effect on whether the patient feels that they are listened to and empowered to ask questions and discuss their preferences and can open up opportunities to make informed choices (O)	[31, 35, 40, 43, 49, 51, 52]
	17	When HCPs adapt their communication styles (including timing and content of information) to meet patient's needs and also recognise that these needs may change over time (C), then patients receive information at a time that is appropriate for them (and carers) and are able to ask questions on their own terms (M). Then they feel better informed and ready to become involved in treatment decisions (O)	[40, 53, 56]
	18	If patients are given a communication aid (question prompt list) to aid consultations but consultations are not tailored to meet their needs (C), they can still feel unprepared for a role in discussions about end‐of‐life prognosis of (M) as they may not necessarily request or discuss information about prognosis or become actively involved in the shared decision‐making process (O)	[42, 43]
	19	If a communication aid (e.g., consultation planning tool) includes a tool to plan questions in consultations and/or a tool to plan and/or record discussions in consultations (C), then nurses can support patients with questions and the consulting HCP can provide more clear answers to patients. These provide them with more personalised care (O)	[46, 47]
Involvement of palliative care specialists and inclusion of palliative care information and concepts	20	When HCPs talk to patients about their role in symptom and pain management (palliative care) and avoid presenting unrealistic hope or palliative care specialists are involved in delivering decision aids and in discussions with patients about treatment options (C), then it can help inform patients about palliative care and familiarise them with appropriate terminology and improve their understanding of the meaning and purpose of palliative care (M). This can support them in reflecting on uncertainties and making appropriate decisions and they may accept palliative care as a viable option to active treatments (O)	[50, 56, 57, 58, 62, 63]

Abbreviation: HCP, healthcare professionals.

### 3.2 Develop a narrative and make recommendations (step 7)

#### 3.2.1 Programme theory development (step 7)

We expanded the initial programme theory, based on our findings, to build an overarching explanation of the relationship between the range of different contexts, mechanisms and outcomes and to inform future intervention design. We integrated the conceptual framework by Kim et al.^27^ to enable us to reflect on how patients (and family) can be supported through the process of engaging with information, decision‐making and reflecting on their experiences and decisions, while at the same time also considering future options and decisions.

**Figure 3:**
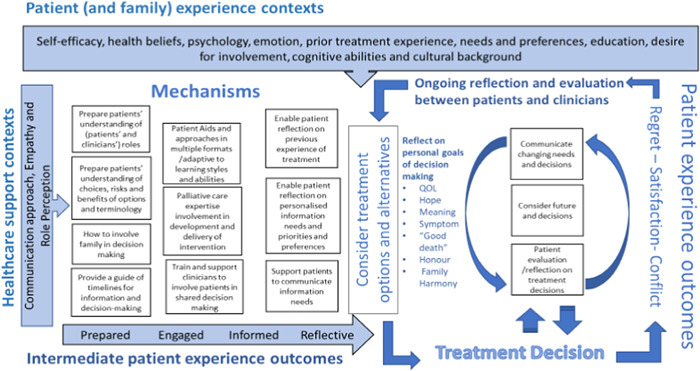
Programme theory to support patients with advanced cancer make decisions about treatment and palliative care.

Our final programme theory (Figure 3) demonstrates how contexts relating to patients and healthcare practitioners can interact with a range of mechanisms to help patients become prepared, engaged, informed and reflective throughout their decision‐making experiences throughout the disease trajectory. We have mapped which theories in Table 3 help inform the programme theory.

### 3.3 Patient and clinician's contexts

We include contexts relating to patient' characteristics: capacity, cultural background, self‐efficacy, health beliefs, psychology, emotions, prior experience of cancer treatment, desire for involvement in treatment decision‐making and their level of cognition (theories 1–4 and 6). HCP support contexts include communication approaches (including their attitudes towards SDM, communications styles, terminology) (theories 15–17 and 20), the provision of empathy (theory 16) and role perception (theory 15) within the particular decision‐making context.

### 3.4 Mechanisms for supporting patient's engagement with treatment decision‐making

#### 3.4.1 Preparation

Patients need to understand that there will be a choice involved when discussing the risks and benefits of treatments and to be clear about their and the clinician's roles in treatment decision making (theory 1). Patients also need time to be prepared and planned for conveying their information needs and communication preferences to HCPs and engaging with the terminology that will be used (theory 19). Preparation time may also be useful to support patients by speaking with family and involving them in their decision‐making (theory 11). Additionally, patients could benefit from a guide of timescales associated with information exchange and treatment decision‐making (theories 10–14).

#### 3.4.2 Providing information and decision support

Providing patient aids in clear and multiple formats helps patients understand their options and engage in decisions (theories 7–9). Including palliative care and prognostic information and terminology in patient aids and involving palliative care specialists in developing patient aids and in discussions with patients to support patients with decision making about active treatment or palliative care, also helps patients make more informed decisions (theory 20). Providing training to HCPs on using SDM approaches is also essential to decision support (theory 18).

#### 3.4.3 Reflection on personal needs, preferences and prior treatment experience

Supporting patients to reflect on previous treatment when engaging with treatment decision‐making and helping them understand differences in side effects and tolerance to treatment could help them understand the contextual differences when a new decision is presented. Enabling reflection on patients' personal information needs and preferences and communicating these to HCPs in consultations is essential in considering treatment and alternatives (theories 3, 4, 10 and 11).

#### 3.4.4 Ongoing reflection and evaluation of treatment experience

We include further reflection and evaluation of prior treatment experiences and decisions (theories 3 and 4). We also include mechanisms related to awareness and consideration of future decisions and the opportunity for patients to reflect on and communicate their changing needs and decisions.

#### 3.4.5 Treatment decision

Treatment decisions are not necessarily definitive in the programme theory, they are made and can be reflected on and changed or renegotiated with the support of HCPs.

### 3.5 Patient experience outcomes

Intermediate outcomes for patients are that they are better prepared for engaging with information and treatment decision making. They should also be then better engaged and informed and able to reflect on their personal goals of decision‐making before making a treatment decision. The outcomes of their decision can then result in regret, satisfaction or conflict (theories 5, 7, 8 and 11).

Subsequently, we presented and discussed inferences made in the programme theory to our public involvement representatives and stakeholders to ensure their perspectives were included in the programme theory and ask for their perspectives on the importance of the finding and the ways findings could be used to influence changes in policy and practice. It was noted that there could be an opportunity for patients to be more prepared to ask questions and have patient‐centred conversations. Stakeholders also referred to a need to understand hcp motivations to embrace changes in communication styles. We were reminded by our PPI representatives to ensure that our programme theory and recommendations took into account family members also present in consultations. Together with our stakeholders, we developed ideas for intervention components that might be successful for different cancer patients and in different settings, that is, cancer clinics/appointments) [Stakeholder/PPI involvement meeting 4].

## 4 DISCUSSION

### 4.1 Principal findings

Our review findings show the range of contexts and mechanisms that can potentially improve patients' experiences of treatment decision making when considering treatment options for advanced noncurative cancer. These include clinician's positive attitude and behaviour towards SDM; patients' skills in engaging with information and reflections on prior treatment experiences; the content and format of patient decision aids (balanced information about treatment options, multiple formats, palliative care terminology explained); involvement from palliative care specialists and the timing of delivery and the time provision before decision making. Positive outcomes for patients included understanding information; acceptability of using a decision aid; ability to participate in SDM; the level of control they feel they have in the decision‐making process and whether they feel less conflict and more satisfaction regarding their decision.

### 4.2 Findings within the context of other literature

The findings illustrate that using appropriately designed decision support tools can improve knowledge about options, risks and outcomes, and also reduce decisional conflict and regret for patients, reflecting previous studies.^63^, ^64^ We identified a limited number of relevant decision support aids (nine) that specifically focus on the treatment decisions for advanced non‐curative cancer and provide information regarding palliative care. This reflects the findings of a wider research theme which highlights a lack of information and decision support provided to patients about supportive or palliative care when considering end‐of‐life treatment options.^60^, ^65^ Our findings supported other evidence that incorporating palliative care expertise early in the patient's pathway can increase patients' understanding and consideration of the best supportive care and palliative care options.^66^, ^67^

We found that multi‐component (e.g., audio and visual) decision support interventions delivered by HCP are associated with mechanisms that lead to positive outcomes in supporting patients to understand and engage in SDM. This reflects previous research that emphasises the need for decision aids to be accompanied by HCP advice and support during the SDM process^20^ and throughout the patient's disease journey.^68^ It is also essential that patients' willingness and readiness to participate in SDM are considered when HCPs engage patients in discussions about treatment and care options, as patients' individual needs will vary.^69^ Patient decision aids can encourage patients and caregivers to be partners in decision making and support HCP to elicit patients' goals and values.^70^ Decision support tools and approaches need to be adaptable to ensure patients' varying cultural backgrounds, needs and preferences are supported, particularly where patients have additional educational needs^62^ and varying cognitive abilities.^68^

Previous studies have provided evidence that Internet/digital delivery can provide the right information (rapidly updated), to the right person (tailored), at the right time (the appropriate point in the decision‐making process).^71^ However, only a few relevant studies in this review explored the use of digital support tools with patients with advanced cancer and therefore changes in how people interact with technology for decision making should be considered when designing an intervention.^72^, ^73^

### 4.3 Policy and practice implications

The context‐focused findings of our review can inform policies, strategies and guidance, relating to SDM and patient involvement.^74^, ^75^ Currently, there is no one particular model or decision support tool for this patient cohort that adequately addresses the variety of patients' needs and contexts at this stage in their cancer pathway. It was clear from our findings that for patients with advanced cancer, making decisions about active palliative treatment and palliative care appropriate information is required at a *time which is suitable and personalised to their individual needs*, and is accessible in multiple formats. Patients can benefit from the information provided through decision support tools and discussions with HCPs that are tailored to their prognosis and explain the benefits and harms of treatment options.^76^, ^77^

Patients benefit from honest and open discussions regarding their individual treatment outcomes and need balanced and understandable information regarding their treatment options.^75^, ^78^ Ensuring that the goals and preferences of patients are incorporated into discussions regarding healthcare, using the best available balanced evidence aligns with Value‐Based Healthcare strategies.^76^, ^77^ Our findings suggest that HCPs should also allow time for patients to engage with decision support and communication tools outside of consultations and enable patients to later reflect on and re‐evaluate decisions and discuss changes to their treatment plan. This goes beyond the steps taken in a single SDM event^20^ Giving patients access to written summaries of consultations and consultation planning tools could help support reflection of treatment decisions in addition to treatment and advanced care planning.^74^, ^79^, ^80^

Our review highlights that palliative care specialists should be involved in the decision support process (including the development of decision and communication tools), integrating palliative care support more consistently within the patient pathway can lead to improvements in quality of life; quality of death and potentially slow down cancer progression and prolong length of life.^78^, ^79^, ^80^, ^81^, ^82^

### 4.4 Strengths and limitations

The review provided insights into what works when and for who in relation to interventions for advanced cancer. Stakeholder involvement provided in‐depth practical knowledge and experience throughout the realist review process, which provided regular opportunities for reflection and refinement of the theories relating to first‐hand experiences.

Including PPI representatives in stakeholder meetings (in line with the UK national standards for public involvement in research (see http://nihr.ac.uk/pi-standards/home)^83^ and using GRIPP2^84^ to ensure appropriate inclusion and reporting has enhanced the findings of the review and helped us build our programme theory. HCPs and PPI involvement in developing recommendations for policy and practice and future research helped us ensure that these were relevant and practical.

Applying the conceptual framework helped explain how decision‐making across the end‐of‐life trajectory is a cyclical, iterative process and experiences with decision processes or outcomes at one point in the trajectory may inform future decisions. This enabled us to consider a more personalised approach to informed and SDM processes which includes reflection on past decisions.

Most of the relevant studies of decision support tools were early or feasibility studies and so required further testing. So, there are limitations to understanding how they would work in routine clinical settings.

### 4.5 Future research/intervention development

Further research is needed to develop interventions to support patients with advanced cancer to make fully informed decisions about treatment and palliative care options, as there are currently no decision‐support tools or processes that fully address the needs and supports of these patients. The varying contexts relating to patient's, caregivers and family experiences need to be considered when designing and implementing such interventions. Involving HCPs who work in advanced cancer care, palliative care, patients and public involvement representatives and service users to co‐produce the design of the intervention, will help ensure that any intervention is suitable for application within healthcare settings and useful for patients.

A multi‐component, multiple‐format and multistage intervention based on the programme theory produced in this review could address requirements for different information formats and tools to support patients and enable the delivery of time‐sensitive information and facilitate time‐sensitive decisions. It is clear from our review findings and other reviews^27^ that there needs to be reflection and evaluation of past decisions and an understanding of how they influence future decisions. Patients and family caregivers may have made similar decisions along their cancer treatment trajectory but need to understand nuances involved in decision‐making events as they arise (i.e., changes in patient‐related contexts and potential outcomes). These are components that current decision aids and communication aids do not include, and future research and development could test the feasibility and acceptability of a tool to support this. A mixed methods realist evaluation approach could be used to develop and evaluate a complex intervention and seek to assess what is feasible and acceptable for whom and under what circumstances.^26^, ^85^

## 5 CONCLUSIONS

The review findings illustrate key contexts and mechanisms that influence decision‐making outcomes for patients with advanced incurable cancer. This includes HCPs' approaches towards SDM and patients' health literacy skills and prior experiences; the content and format of the patient decision aid, as well as the timing of delivery and the time provision before decision making and the need for palliative care specialists to be involved in the design and implementation of an intervention at the earliest possible stage. A co‐produced approach to intervention design involving oncology and, palliative care HCPs, patients and their families could ensure patients are appropriately informed and that the intervention mechanisms are suitable to address their needs and positively enhance their experience.

## AUTHOR CONTRIBUTIONS

Michelle Edwards lead the study. Michelle Edwards, Daniella Holland‐Hart and Annmarie Nelson conceived the idea of the study. Michelle Edwards and Daniella Holland‐Hart collected, analysed and wrote up the results and drafted the manuscript. Mala Mann developed and conducted the searches. Public contributors (Kathy Seddon and Peter Buckle) supported the study and carried out the validation of the findings. All authors reviewed and commented on the drafts of this manuscript. All authors read and approved the final manuscript.

## CONFLICT OF INTEREST STATEMENT

The authors declare no conflict of interest.

## Supporting information

Supporting information.

Supporting information.

Supporting information.

Supporting information.

Supporting information.

## Data Availability

The data that support the findings of this study are available from the corresponding author upon reasonable request.
