# Supplementary material for: Understanding how shared decision‐making approaches and patient aids influence patients with advanced cancer when deciding on palliative treatments and care: A realist review
Source: Health Expect. 2023 Jul 13;26(6):2109–26. doi: 10.1111/hex.13822 (PMC10632651; doi:10.1111/hex.13822)
Supplement: Supplementary file 1 — Supporting information. [file HEX-26--s004.pdf]

Appendix 1: List of key papers used to develop initial theories

Theme	Explanatory account
Decision Aids/ communication tools	Joseph-Williams N, Abhyankar P, Boland L, Bravo P, Brenner AT, Brodney S, Coulter A, Giguere A, Hoffman A, Körner M, Langford A, Légaré F, Matlock D, Moumjid N, Munro S, Dahl Steffensen K, Stirling C, van der Weijden T. What Works in Implementing Patient Decision Aids in Routine Clinical Settings? A Rapid Realist Review and Update from the International Patient Decision Aid Standards Collaboration. Med Decis Making. 2020 Dec.<br><br>Krist, AH, Woolf, SH, Hochheimer, C, et al. Harnessing information technology to inform patients facing routine decisions: cancer screening as a test case. Ann Fam Med. 2017;15(3):217–24.
	Joseph-Williams N, Abhyankar P, Boland L, Bravo P, Brenner AT, Brodney S, Coulter A, Giguere A, Hoffman A, Körner M, Langford A, Légaré F, Matlock D, Moumjid N, Munro S, Dahl Steffensen K, Stirling C, van der Weijden T. What Works in Implementing Patient Decision Aids in Routine Clinical Settings? A Rapid Realist Review and Update from the International Patient Decision Aid Standards Collaboration. Med Decis Making. 2020 Dec.
	Nelson A, Longo M, Byrne A, et al. Chemotherapy decision-making in advanced lung cancer: a prospective qualitative study BMJ Supportive & Palliative Care Epub ahead of print: 2020;0:1–7. doi:10.1136/ bmjspcare-2020-002395
	Joseph-Williams N, Abhyankar P, Boland L, Bravo P, Brenner AT, Brodney S, Coulter A, Giguere A, Hoffman A, Körner M, Langford A, Légaré F, Matlock D, Moumjid N, Munro S, Dahl Steffensen K, Stirling C, van der Weijden T. What Works in Implementing Patient Decision Aids in Routine Clinical Settings? A Rapid Realist Review and Update from the International Patient Decision Aid Standards Collaboration. Med Decis Making. 2020 Dec.
	Leighl, N., et al. (2011) Supporting treatment decision making in advanced cancer: a randomized trial of a decision aid for patients with advanced colorectal cancer considering
Clinician's communication and decision support approaches	Nelson A, Longo M, Byrne A, et al. Chemotherapy decision-making in advanced lung cancer: a prospective qualitative study BMJ Supportive & Palliative Care Epub ahead of print: 2020;0:1–7. doi:10.1136/ bmjspcare-2020-002395

	Nelson A, Longo M, Byrne A, et al. Chemotherapy decision-making in advanced lung cancer: a prospective qualitative study BMJ Supportive & Palliative Care Epub ahead of print: 2020;0:1–7. doi:10.1136/ bmjspcare-2020-002395
	Beck, AC, Ellington, L, Mooney, K et al . Making treatment decisions at end of life in a comprehensive cancer center. Journal of Clinical Oncology 34, no. 26_suppl (October 09, 2016) 51-51.
	Nelson A, Longo M, Byrne A, et al. Chemotherapy decision-making in advanced lung cancer: a prospective qualitative study BMJ Supportive & Palliative Care Epub ahead of print: 2020;0:1–7. doi:10.1136/ bmjspcare-2020-002395
	Leighl, N., et al. (2011) Supporting treatment decision making in advanced cancer: a randomized trial of a decision aid for patients with advanced colorectal cancer considering chemotherapy. Journal of clinical oncology 29, 2077-2084 DOI: 10.1200/JCO.2010.32.0754
	Chen JJ, Roldan CS, Nichipor AN, Balboni TA, Krishnan MS, Revette AC, Hertan LM, Chen AB. Patient-Provider Communication, Decision-Making, and Psychosocial Burdens in Palliative Radiotherapy: A Qualitative Study on Patients' Perspectives. J Pain Symptom Manage. 2021 Feb 5:S0885-3924(21)00151-2. doi: 10.1016/j.jpainsymman.2021.01.129. Epub ahead of print. PMID: 33556491.
	(Stakeholder PB)
Patient preference	Bergqvist, J. and P. Strang (2017). "The will to live-breast cancer patients perceptions' of palliative chemotherapy." Acta Oncologica 56(9): 1168-1174.
	Nelson A, Longo M, Byrne A, et al. Chemotherapy decision-making in advanced lung cancer: a prospective qualitative study BMJ Supportive & Palliative Care Epub ahead of print: 2020;0:1–7. doi:10.1136/ bmjspcare-2020-002395
