# Supplementary material for: Understanding how shared decision‐making approaches and patient aids influence patients with advanced cancer when deciding on palliative treatments and care: A realist review
Source: Health Expect. 2023 Jul 13;26(6):2109–26. doi: 10.1111/hex.13822 (PMC10632651; doi:10.1111/hex.13822)
Supplement: Supplementary file 2 — Supporting information. [file HEX-26--s002.pdf]

Appendix 2: Ovid Meline search strategy

Database: Ovid MEDLINE(R) ALL <1946 to April 06, 2021>
Search Strategy:

----

1 Decision Making/ (98039)

2 Decision Support Techniques/ (21055)

3 ("Communication tool" or "Patient preference tool" or "Question prompt tool" or "Question prompt list").tw. (1090)

4 (decision* adj4 (support* or aid* or tool* or instrument* or technolog* or technique* or system* or program* or algorithm* or process* or method* or intervention* or material*)).tw. (85716)

5 "shared decision making".tw. (8921)

6 (tools adj3 support communication).tw. (10)

7 "decision making process".tw. (14737)

8 treatment decision*.tw. (21313)

9 (interacti* adj4 (tool* or aid)).tw. (5410)

10 "Patient Preference"/ (9237)

11 treatment decision*.tw. (21313)

12 (Shared decision making adj3 (tools or process or model*)).tw. (1167)

13 Holistic needs assessment.tw. (45)

14 ("Information tool*" or "information support*" or "communication support").tw. (2161)

15 (Patient diary or preference elicitation or Treatment summary).tw. (664)

16 or/1-15 (216784)

17 ("Palliative Care" or "supportive care").tw. (45381)

18 ("last year of life" or LYOL or "end of life" or "end of their lives").tw. (24658)

19 ("Terminal Care" or "hospice care").tw. (4634)

20 ("terminal* ill*" or " terminal stage" or "advanced stage ill*").tw. (9572)

21 ("last six months of life" or "last 6 months of life").tw. (272)

22 (end of life or EOL).tw. (24378)

23 or/17-22 (72688)

24 ((Advanced or terminal or non-curative) adj4 (carcinoma* or adenocarcinoma or malignan* or neoplasm* or cancer* or tumo?r*)).tw. (124434)

25 metastatic.tw. (238522)

26 Neoplasms/ and (Advanced or terminal or non-curative).mp. (24123)

27 or/24-26 (352090)

28 16 and 23 and 27 (855)

29 (decision making adj3 (oncology or cancer or palliative)).tw. (1718)

30 ("decision aids" and ("advanced cancer" or end of life)).tw. (48)

31 (Support adj Shared Decision Making).mp. and Palliative.tw. (7)

32 ("treatment decision making" and "advanced cancer").tw. (33)

33 ("decision making" and "advanced cancer").tw. (385)

34 or/29-32 (1799)

35 28 or 34 (2597)

36 limit 35 to (english language and humans and yr="2000 - 2021") (1999)
