# Supplementary material for: Understanding how shared decision‐making approaches and patient aids influence patients with advanced cancer when deciding on palliative treatments and care: A realist review
Source: Health Expect. 2023 Jul 13;26(6):2109–26. doi: 10.1111/hex.13822 (PMC10632651; doi:10.1111/hex.13822)
Supplement: Supplementary file 3 — Supporting information. [file HEX-26--s005.pdf]

**Appendix 3: Abstract and full paper screening tool**

| | |
|---|---|
| 1 - Highly Relevant | Relates to the use of decision aids in clinical consultations with adults with advanced cancer for decisions about treatments (chemotherapy, radiotherapy, immunotherapy) vs supportive care<br>OR<br>Relates to the use of shared decision making in clinical consultations with patients with advanced cancer making for making treatment decisions about chemotherapy, radiotherapy, immunotherapy vs supportive care<br>OR<br>Relates to any decision aid that adults with advanced cancer use independently (outside clinical consultations) or with family/friends/carers to support making decisions about treatments (chemotherapy, radiotherapy, immunotherapy) vs supportive care |
| 2 – Probably Relevant | Relates to the use of any kind of communication tools that are used in clinical consultations with or by adults with advanced cancer to support information sharing and decision making about chemotherapy, radiotherapy, and immunotherapy vs supportive care<br>OR<br>Relates to any kind of communication tools that adults with advanced cancer use 'independently' (outside clinical consultations) to support information sharing and decision making about chemotherapy, radiotherapy vs supportive care. |
| 3 – Possibly Relevant | Relates to seeking information and support for treatment decision making for advanced cancer.<br>OR<br>Relates to decisions aids/ communication tools used with adults with advanced cancer regarding preferences and priorities for treatment. |
| 4 – Likely irrelevant | Does not meet above criteria |
