# Supplementary material for: Understanding how shared decision‐making approaches and patient aids influence patients with advanced cancer when deciding on palliative treatments and care: A realist review
Source: Health Expect. 2023 Jul 13;26(6):2109–26. doi: 10.1111/hex.13822 (PMC10632651; doi:10.1111/hex.13822)
Supplement: Supplementary file 4 — Supporting information. [file HEX-26--s003.pdf]

Appendix 4: Data extraction template

Number:

Title:

Author (Year):

**Study Design**	
**Aim of study**	
**Patient group**	
**Consultation context**	
**DA/ intervention type**	
**DA objective**	
**Delivery**	
**Measures**	
**Data collection**	
**Key findings**	
**Strengths**	
**Weaknesses**	

Evidence levels – 1a quantitative primary data 1b qualitative primary data 2 – author interpretation, 3 a reviewer interpretation of literature 3b reviewer interpretation based on their clinical experience

If	Then	Because	Evidence Level
